# Mixed Genotype Infections with Hepatitis C Virus, Pakistan

**DOI:** 10.3201/eid1708.100950

**Published:** 2011-08

**Authors:** Sadia Butt, Muhammad Idrees, Irshad Ur Rehman, Haji Akbar, Muhammad Shahid, Samia Afzal, Saima Younas, Iram Amin

**Affiliations:** Author affiliation: University of the Punjab, Lahore, Pakistan

**Keywords:** hepatitis C virus, viruses, mixed genotype infections, prevalence, Pakistan, letter

**To the Editor:** The prevalence of hepatitis C virus (HCV) infection is high (8% of the population) in Pakistan (*1*). HCV is an RNA virus that has a high mutation rate. This high rate results in extensive genetic heterogeneity, and HCV isolates are found as either quasispecies or genotypes (*2*). Humans can be co-infected with >1 genotype (mixed genotype infection) of this virus (*3*). The rate of HCV mixed genotype infections is extremely variable for different regions and for the same group of patients tested by using different assays (*4*). Thus, it is difficult to determine the prevalence of mixed genotype infections by currently available assays, including direct DNA sequencing, because they are designed to identify only the HCV genotype dominant in that particular population. Consequently, genotypes present at lower frequencies could be missed or mistyped (*5*).

To determine the prevalence of HCV mixed genotype infections, we retrospectively analyzed genotyping data for paired serum samples from 22,125 HCV-infected patients during the past 11 years (March 2000–May 2010) for all regions in Pakistan by using molecular-based genotype-specific methods (*6**,**7*). A total of 12,036 (54.4%) were male patients and 10,089 (45.6%) were female patients.

The sensitivity and reliability of the assay we used has been assessed and found to be superior to restriction fragment length polymorphism analysis and serotyping methods for detection of mixed genotypes in a viral population. Our method can detect a small amount (8.3%) of HCV RNA in a mixed genotype population (*7*). Restriction fragment polymorphism analysis can detect 2 genotypes only if 1 of them represents >41.6% of the genotypes in a mixed genotype population.

Of 22,125 HCV RNA-positive serum samples, type-specific PCR bands were observed in 18,181 (82.2%) samples and 3,944 (17.8%) were not typeable. A total of 1,007 (5.5%) patients had HCV mixed genotype infections.

The distribution of mixed genotype infections in 1,007 patients is shown in Figure A1. Infection with mixed genotype 3a + 3b was most prevalent (43.79%). Age distribution of patients with mixed genotype infections is shown in the Table. Approximately 33% of patients with mixed genotype infections were 31–40 years of age and 22.5% were 41–50 years of age.

**Table:** Distribution of mixed genotype infections with hepatitis C virus in 1,007 patients, by age, Pakistan, March 2000–May 2010

Genotype	Mixed genotype infection	Patient age, y								Total no. infections
		0–10	11–20	21–30	31–40	41–50	51–60	>60	Unknown	
1	1a + 1b	0	0	2	5	6	7	0	1	21
1 + 2	1a + 2a	0	0	2	1	2	0	0	0	5
	1b + 2a	0	0	0	1	2	1	0	1	5
	1b + 2b	0	0	1	0	0	0	0	0	1
1 + 3	1a + 3b	0	3	4	4	3	1	0	0	15
	1a + 3a	0	4	29	51	26	16	2	17	145
	1c + 3a	0	1	2	2	1	0	0	1	7
	1b + 3b	0	2	3	2	1	0	0	0	8
	1b + 3a	0	7	45	90	79	32	3	31	287
1 + 4	1a + 4	0	0	0	0	3	1	0	1	5
2 + 3	2a + 3a	0	2	5	9	4	3	0	1	24
	2a + 3b	0	1	5	8	5	3	0	1	23
	3a + 2b	0	0	1	2	2	0	0	1	6
3	3a + 3b	1	15	94	142	87	46	13	43	441
3 + 4	3b + 4	0	1	0	2	2	1	0	0	6
	3a + 4	0	0	0	2	4	0	0	0	6
3 + 6	3a + 6a	0	0	0	1	0	1	0	0	2
Total		1	36	193	322	227	112	18	98	1,007

Patterns of HCV mixed genotype infections in Pakistan are similar to those reported from India and Iran (*8*). However, the prevalence of HCV mixed genotype infections was lower (2%) (*8*) for Iran than for Pakistan. This lower rate may have been caused by use of a genotyping kit that can detect only genotypes 1a, 1b, 2, and 3a. Thus, mixed infections with other genotypes would not have been detected. A recent study in Brazil reported that mixed genotype infections were detected in 3.9% of intravenous drug users and 7.1% of former injecting drug users (*9*). These rates were similar to those in our study. In contrast, data from Sweden and Russia showed no mixed genotype infections in serum samples of chronically infected intravenous drug users, hemodialysis patients, and patients with hemophilia (*10*).

Women (288/7,390, 3.89%) in Pakistan had significantly fewer HCV mixed genotype infections than men (719/10,791, 6.66%) (p<0.01). This finding might be the result of women having fewer risk factors for contracting mixed genotype infections. Possible risk factors for infection with mixed genotype infections analyzed were blood transfusions and use of blood products (51.3%); multiple use of needles or syringes (18.4%); sharing razors during shaving or circumcision, piercing instruments, nail clippers, and toothbrushes (13.7%); and major or minor dental surgery (9.5%). Mode of transmission was not clear for 7.1% of the patients.

In conclusion, the prevalence of HCV mixed genotype infections in Pakistan is higher than previously reported and higher among men (p<0.01). Comprehensive and detailed investigations are warranted to evaluate the clinical role of chronic HCV mixed genotype infections, provide essential information that can be used to determine type and duration of therapy needed, and predict disease outcome.

## Figures and Tables

**Figure A1:**
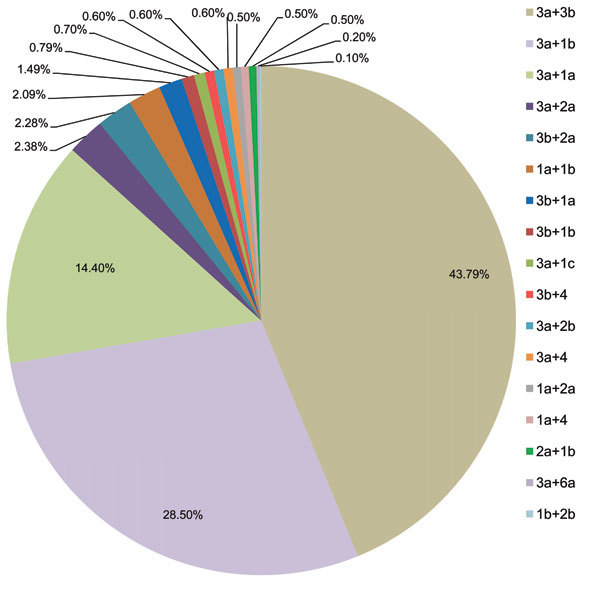
Frequency of mixed genotype infections with hepatitis C virus in 1,007 patients, Pakistan, March 2000–May 2010.
